# First description of bacterial and fungal communities in Colombian coffee beans fermentation analysed using Illumina-based amplicon sequencing

**DOI:** 10.1038/s41598-019-45002-8

**Published:** 2019-06-19

**Authors:** Ana C. de Oliveira Junqueira, Gilberto V. de Melo Pereira, Jesus D. Coral Medina, María C. R. Alvear, Rubens Rosero, Dão P. de Carvalho Neto, Hugo G. Enríquez, Carlos R. Soccol

**Affiliations:** 1 Department of Bioprocess Engineering and Biotechnology, Federal University of Paraná (UFPR), 19011 Curitiba, Paraná, 81531-980 Brazil; 2 Department of Process and Biotechnology, Mariana University, 520002 Pasto Nariño, Colombia

**Keywords:** Food microbiology, Environmental biotechnology

## Abstract

In Colombia, coffee growers use a traditional method of fermentation to remove the cherry pulp surrounding the beans. This process has a great influence on sensory quality and prestige of Colombian coffee in international markets, but has never been studied. Here we use an Illumina-based amplicon sequencing to investigate bacterial and fungal communities associated with spontaneous coffee-bean fermentation in Colombia. Microbial-derived metabolites were further analysed by high–performance liquid chromatography and gas chromatography–mass spectrometry. Highly diverse bacterial groups, comprising 160 genera belonging to 10 phyla, were found. Lactic acid bacteria (LAB), mainly represented by the genera *Leuconostoc* and *Lactobacillus*, showed relative prevalence over 60% at all sampling times. The structure of the fungal community was more homogeneous, with *Pichia nakasei* dominating throughout the fermentation process. Lactic acid and acetaldehyde were the major end-metabolites produced by LAB and *Pichia*, respectively. In addition, 20 volatile compounds were produced, comprising alcohols, organic acids, aldehydes, esters, terpenes, phenols, and hydrocarbons. Interestingly, 56 microbial genera, associated with native soil, seawater, plants, insects, and human contact, were detected for the first time in coffee fermentation. These microbial groups harbour a remarkable phenotypic diversity and may impart flavours that yield clues to the *terroir* of Colombian coffees.

## Introduction

The coffee plant is native to tropical Africa and southern Asia. It was introduced to the Americas in the late 18^th^ where, currently, the largest producing countries are located, including Brazil, Colombia, Peru, Guatemala, Honduras, and Mexico. Colombia is the third-largest coffee producer in the world after Brazil and Vietnam^1^. Colombian coffee comes almost exclusively from Arabica cultivars and is often regarded as some of the highest-quality coffee in the world. This is mainly due to rich volcanic-ash soil, abundant annual rainfall (200 mm per month on average), and high altitudes (1,800 to 2,000 m) where the coffee trees are grown^2^.

In the international market, coffee is classified into two main categories according to the postharvest processing technology used to remove the outer layers adhered to the fruits: ‘natural coffee’, produced from coffee beans processed on the farm by the simple method of sun-drying, known as dry processing; and ‘washed coffee’, produced from coffee beans that undergo a relatively complex series of steps, including depulping, fermentation, and sun-drying, known as wet processing^3,4^. The abundant rainfall and warm temperatures in the regions of Colombia where coffee is grown causes an immediate, undesirable fermentation after harvest^4^. The most practical way to avoid this unwanted fermentation is the wet processing method, whereby fermentation can be controlled in terms of time, temperature, and exchange of water; spontaneous development of microorganisms can be better managed to minimize any adverse impacts on coffee quality^4^.

Due to the high quality of ‘washed coffee’ produced in the Colombia, nowadays, fermentation process is a subject of worldwide interest^3,5^. Countries such as Brazil, Guatemala, Honduras, and Ecuador export, annually, tons of coffee beans classified as ‘washed coffee’. Thus, studies have been devoted to uncovering the microbial diversity and chemical changes involved in spontaneous coffee fermentation worldwide^6–8^. Recent microbiome studies have shown the rich microbial diversity, comprising more than 80 genera, in coffee fermentations conducted in Brazil and Ecuador^9,10^. The complex microbial activity produces ethanol, lactic acid, and a range of minor compounds, such as esters, higher alcohols, aldehydes, and ketones, which are speculated to diffuse into the beans and impact in the final composition of coffee beverage^11–15^.

Here we conducted a comprehensive study of the microbial structure during spontaneous coffee-bean fermentation in Colombia. By sampling the fermentation process at different times, we were able to describe the composition, distribution, and dynamics of naturally occurring microbial communities. Our study describes, for the first time, the great diversity of bacteria and yeasts harboured in the most traditional method of coffee fermentation in the world. These core microbiomes have strong influence on the metabolites produced during the fermentation and may impact the chemical composition of Colombian coffees.

## Results and Discussion

We obtained a total of 297,126 and 129,126 300-bp quality-filtered 16S and 18S rRNA gene sequences from temporal sampling, respectively. The alpha rarefaction curves of each temporal analysis suggest that most of the microbial diversity has been sampled (Supplemental Fig. S1). Estimated richness (Ace and Chao) and diversity (Shannon and Simpson) were calculated to evaluate the alpha and beta diversity of microbial communities through the fermentative process (Table 1). The higher ACE and Chao index indicated the higher microbial richness, which means that richness of microbiota increased during the fermentation process. On the other hands, diversity depends not only on richness, but also on evenness. Thus, due to the gradual increased of read sequences relative to LAB species and decreased evenness, Simpson and Channon index decreased in the course of the fermentation process.

**Table 1:** Microbial community richness and diversity indices of the 16S and 18S rRNA gene sequences for clustering at 97% sequence similarity from Colombian coffee beans fermentation.

Sample (Fermentation time)	Bacteria indices				Fungi indices			
	OTU^a^	Chao^b^	Shannon^c^	Simpson	OTU	Chao	Shannon	Simpson
0	107	1240.5	1.82	0.71	6	127.8	1.15	0.58
6	65	1241.9	1.40	0.46	4	152.9	0.66	0.42
12	82	568.8	1.28	0.44	3	125.1	0.57	0.33
24	110	1384.6	0.82	0.28	6	115.7	0.75	0.42
36	112	1273.5	0.77	0.29	8	133.6	0.73	0.42
48	129	1493.2	1.00	0.49	7	115.0	0.59	0.30

^a^Operational taxonomic units (OTUs) according to the SILVA database.

^b^Chao richness estimator: the total number of OTUs estimated by infinite sampling. A higher number indicates a higher richness (Chao, 1984).

^c^Shannon and Simpson: index to characterize species diversity based on species richness as well as their relative abundance. A higher value represents more diversity.

**Figure 1:**
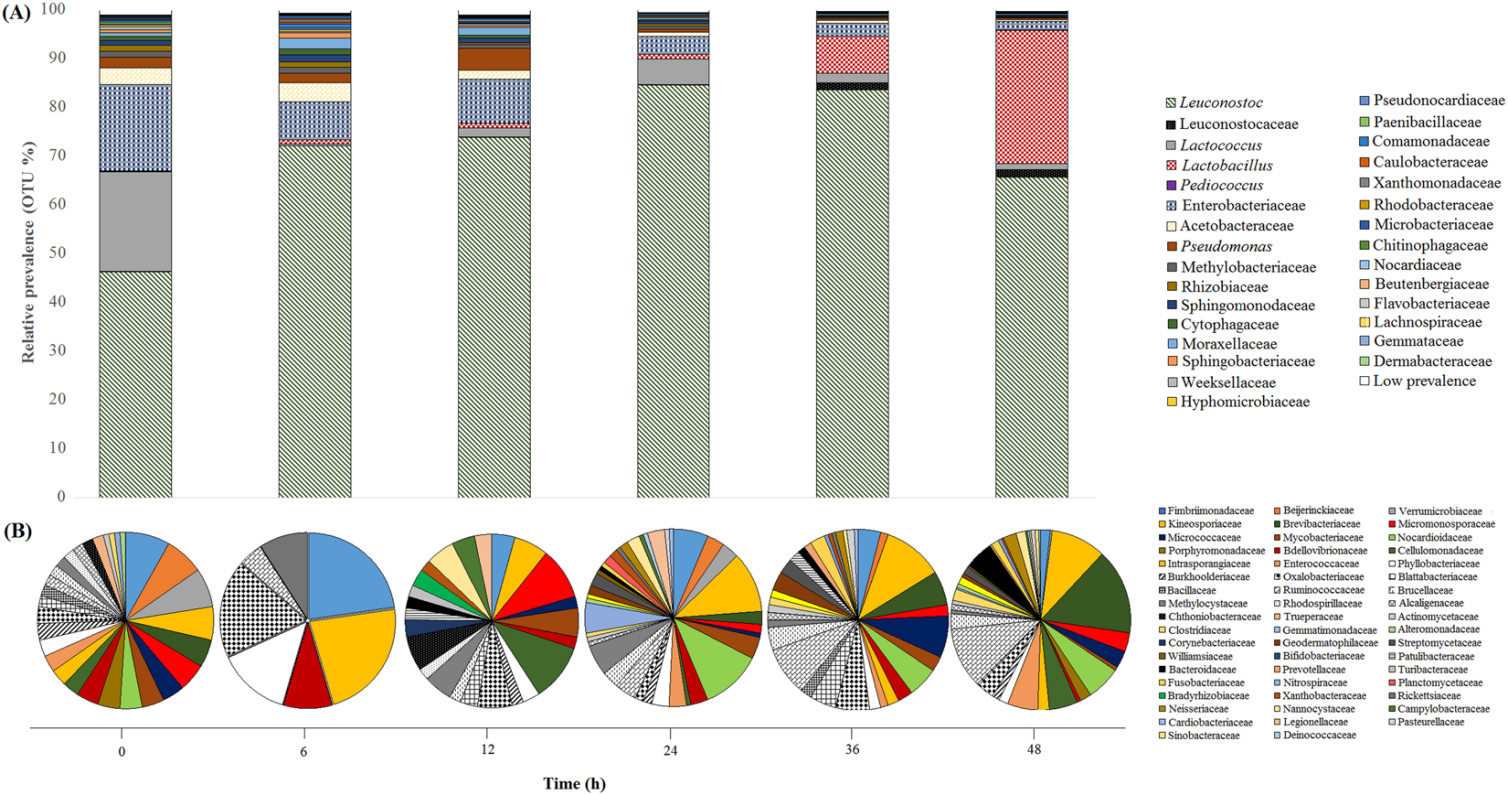
Relative abundance of bacteria at phyla level during Colombian spontaneous coffee beans fermentation process. (**A**) Microbial groups with relative prevalence ≥0.1%. (**B**) Microbial groups with relative prevalence <0.1%. Identification and distribution at genus level are shown in the supplemental material (Table S1).

**Figure 2:**
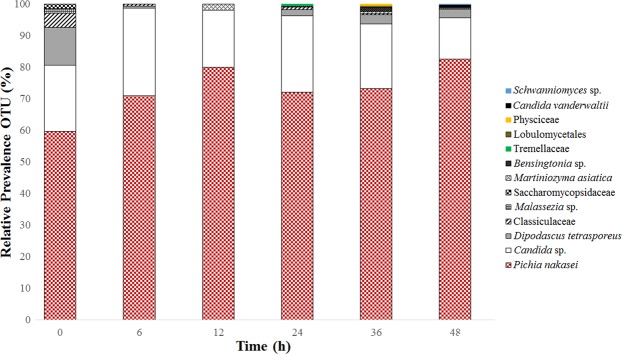
Relative abundance of fungi during Colombian spontaneous coffee beans fermentation process.

The sequences corresponded to 173 different microbial genera after searching in the SILVA database. The complete list of bacteria and fungi at the genus level is shown in the supplemental material (Table S1). This represents a greater diversity than those found in coffee fermentations conducted in Brazil and Ecuador using the Illumina-based amplicon sequencing approach^9,10^. The more microorganisms participate in a community, the more complex the interactions are. As in other well-studied fermentation models, microbial interactions emerge as yeast-bacteria, bacteria-bacteria and yeast-yeast, and also interactions of filamentous fungi with other species^16,17^. Here, *Leuconostoc* in the prokaryote group and *Pichia nakasei* in the eukaryotes were found to govern the fermentation process (Figs 1 and 2). LAB of the genera *Leuconostoc* are commonly found in association with yeast in domestic applications (*e*.*g*., wine, cocoa and kefir) and in nature in over-ripened or faulty fruits^18^. The complex nature of this interaction is highlighted by the observations that (i) the autolysis of yeasts release nutrients, such as amino acids, polysaccharides and riboflavin, favorable for bacterial growth^16,19^, and that (ii) the acidification of the fermentation media by LAB creates a prone environment for yeast development^4^. These positive interactions have been shown to promote desired sensory attributes in wine, sourdough and yogurt. However, information about these mechanisms in coffee fermentation is scarce.

The least-found sequences were commonly associated with microbial groups originating from native soil (Rhizobium, Methylobacterium, Pseudomonas, Bacillus, Burkholderia, Agrobacterium, Azospirilum, Streptomyces, Planctomyces, and Phenylobacterium), water source used in the fermentation (Acinetobacter and Polaromonas), air surrounding the fermentation tank (Pedobacter, Stenotrophomonas, Sphingomonas and Microbacterium), birds and domestic animals (Enterobacter, Micrococcus, Bacillus, Turicibacter, Corynebacterium, and Brevibacterium), insects (Acetobacter, Gluconobacter, Gluconacetobacter, Luteimonas, and Alcaligenaceae), and human related (Sphingobium, Corynebacterium, Malassezia, Candida, Tremellaceae, Enterococcus, Paracoccus, and Caulobacteraceae)^20–30^, of which 54 genera had not been previously reported in the coffee fermentation process (Table S1). Some of these microbial groups harbor a remarkable fermentation profile that includes production of aromatic compounds, enzymes, and organic acids^25,26^. Although present in low proportions, this wide diversity indicate a microbial activity specific to geographical region and niche, and may impart flavours that yield clues to the terroir of Colombian coffees. In addition, this complex ecosystem can be used as a source of microbial and biosynthetic diversity for natural products discovery, such as synthesis of antibiotic, pigment and amino acid, bioremediation of dye- and hydrocarbon-contaminated sites, and accumulation of silver nanoparticles (Supplemental Table S2).

Relative abundance of bacterial groups presenting >0.1% of read sequences is shown in Fig. 1A. The beginning of the fermentation process was characterised by the high prevalence of LAB and bacteria belonging to the families Enterobacteraceae and Acetobacteraceae. The Enterobacteriaceae family was represented by the genera *Erwinia*, *Klebsiella*, *Pantoea*, *Serratia*, *Enterobacter*, and *Citrobacter*, while *Gluconobacter*, *Acetobacter*, *Gluconacetobacter*, *Roseomonas*, and *Roseococcus* genera represented Acetobacteraceae family (Supplemental Table S1). Enterobacteraceae are mainly associated with human contact and formation of off-flavour metabolites, such as 3-isopropyl-2-methoxy-5-methylpyrazine, 2,3-butanediol, and butyric acid^27–29^. Acetobacteraceae are commonly reported in commensal and symbiotic relationship with sugar-rich diet insects, such as bees, *Drosophila*, and ants^30^. As the coffee bean pulp presents high sugar content, Acetobacteraceae can disperse into fermentation sites using the insects as dispersion vectors^31^. The major metabolism of Acetobacteraceae members results in the production of acetic acid—a volatile organic acid that can decrease significantly the coffee beverage quality when found in concentrations higher than 1 g/L^32^. However, members of the Acetobacteraceae and Enterobacteraceae families decreased dramatically after 12 h of fermentation, being overlapped by LAB (Fig. 1A).

Within LAB group, *Leuconostoc* showed a continuous domain of the process with a prevalence peak of 84% observed at 24 h (Fig. 1A). *Leuconostoc* was also a dominant microbial group in coffee fermentations inside Brazil, Mexico, Ecuador, Taiwan, and India biomes^6,9,10,33,34^. This confirms *Leuconostoc* as a core bacterium in coffee-bean fermentation worldwide. In addition, LAB belonging to the families Streptococcaceae (*Lactococcus*) and Lactobacillaceae (*Lactobacillus* and *Pediococcus*) were detected in significant presence during the initial and final fermentation phases, respectively (Fig. 1A). The presence and dominance of LAB indicates that these microorganisms are adapted to the environmental conditions and stressor factors that coffee fermentation imposes, such as low pH, availability of sugars, and competition with other microorganisms^32^. These lactic acid-producing bacteria contribute to the demucilage process of coffee pulp and inhibition of the growth of pathogens, spoilage microbes, and toxin-producing fungi^35^. Other LAB members found in low proportions in this study, such as *Weisselia* and *Fructobacillus*, were reported with significant presence in coffee fermentations of Brazil and Mexico^6,36^. These LAB genera possess specific metabolism, such as improved fructose consumption and extracellular polysaccharides production, which may induce metabolite changes in the Colombian process^37^. Further investigations are necessary to obtain a comparative microbial diversity and activity between different coffee producing regions.

*Pichia nakasei* was the dominant group within eukaryotes, followed by *Candida* sp., *Dipodascus tetrasporeus*, and *Malassezia* sp. (Fig. 2). *Pichia* have been reported as a dominant yeast in coffee beans fermentation conducted in Brazil, Tanzania, and China^7,8,38,39^; however, this is the first study to report the dominance of *P*. *nakasei*. This species have been isolated from fruit or fruit products, and classified into *P*. *kluyveri* clade^40^. Other fungal groups were found at specific time of fermentation process, including *Schwanniomyces*, *Bensingtonia*, *Candida vanderwaltii*, *Martiniozyma asiatica*, Physciceae, Tremellaceae, and *Lobulomycetales* (Fig. 2). Because coffee fermentation take places in an open tank, the process is susceptible to contaminations from human contact, air, insects, and dead leafs^41,42^. In addition, some extremophile yeasts, such as *Dipodascus tetrasporeus* and Lobulomycetales, were for the first time reported in coffee fermentation process. These yeast species are generally associated with deep Pacific Ocean sediment and subglacial sediments of the Andean Mountains^43–45^. The coffee farm sampled in this study is localized at 161 km from Colombian coastal region, which may facilitate the migration of these species into the coffee farm environment.

**Figure 3:**
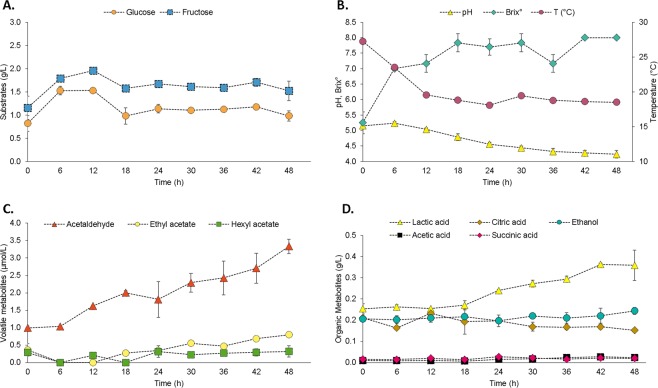
Course of sugar consumption, metabolite formation, pH, temperature and Brix during Colombian spontaneous coffee beans fermentation process.

The changes in major non-volatiles (sugars, organic acids and ethanol) and volatiles (acethaldeyde, ethyl acetate and hexyl acetate) metabolites were quantified in the course of fermentation time (Fig. 3). The initial composition of Colombian coffee pulp was 0.82 g/L glucose, 1.16 g/L of frutocse, 0.20 g/L latic acid, pH 5.2 and 5.3 °Brix. The Brix and sugar concentration showed an increase during the initial 12 h of fermentation (Fig. 3A,B). This observation can be a result of the action of hydrolytic enzymes produced by microbial metabolism, which promotes the breakdown of pectin, cellulose, sucrose, and other coffee pulp complexes carbohydrates, into monomers of glucose and fructose^6,46^. After this increase, both glucose and fructose were partially consumed until 18 h, followed by a stabilization of the concentration until the end of the fermentative process. A residual content of 0.98 and 1.52 g/L for glucose and fructose was observed, respectively. The presence of residual sugars is an evident characteristic of spontaneous coffee fermentations, as previously observed in Brazil, Ecuador, India, and Mexico^6,10,47,48^.

No significant changes were observed in the contents of ethanol, citric acid, acetic acid, and succinic acid (Fig. 3D). On the other hand, lactic acid showed a significant increase, reaching maximum concentration of 0.37 g/L at the end of the fermentation, causing pH decay from 5.2 to 4.2 (Fig. 3B). The steady increase in lactic acid content is correlated to LAB growth that display fermentative metabolism with usually lactic acid as the main metabolic end product^37^. This is also an important end-metabolite associated with coffee fermentation, which assist in the coffee-pulp acidification process without interfere in the product final quality^32,36,49,50^.

The major volatiles measured by GC were acetaldehyde, ethyl acetate, and hexyl acetate (Fig. 3C). Among these, acetaldehyde showed a greater variation through the fermentation, ranging from 0.99 to 3.33 µmol/L. Acetaldehyde is predominantly produced by yeasts of the genus *Pichia*, which have low alcohol dehydrogenase activity responsible for the conversion of acetaldehyde into ethanol^51^. This aromatic molecule is widely known to contribute to fruity sensory notes in alcoholic beverages^52^. The direct relationship between acetaldehyde and coffee quality is, however, not yet known. It is possible to speculate that an intense diffusion during fermentation may have a complementary function in the development of fruity notes of Colombian coffees. Further studies on this relationship are needed

A total of 20 minor volatile metabolites were detected through the fermentative process by GC–MS, including 5 organic acids, 3 alcohols, 1 aldehyde, 4 esters, 2 ethers, 1 furan, 2 hydrocarbons,1 phenol, and 2 terpenes (Table 2). The main metabolites found at the beginning of the fermentation process were 1,2-benzenedicarboxylic acid, 9,12-octadecadienoic acid, and 1-pentadecanal. However, the concentration of these compounds was significantly reduced with the fermentation period, possibly due to evaporation or microbial use as precursors in metabolic routes^3^. On the other hand, the content of 1-hexanol, 2-heptanol, nonanal, isoamyl acetate, 2,2,6-trimethyloctane 2-hydroxy-benzoic acid, linalool, and limonene, which can be formed from microbial activity, increased gradually through the fermentation process. In this respect, LAB and yeasts have a pivotal influence through the generation of different aroma-influencing molecules via central carbon and nitrogen metabolism^3^. Some microbial groups reported in this study, including *Pichia nakasei*, *Candida* sp., *Lactobacillus*, *Lactococcus*, *Leuconostoc*, and *Oenococcus*, are often characterised as important fermenters in the production of wine, cheese, yogurt, dough, and beer due to the production of low-molecular-weight flavour compounds^53–58^. For coffee, however, more studies are necessary to evaluate the direct impact of this compound on the final product quality.

**Table 2:** Concentration of volatile compounds (Area *10^5^) formed during Colombian spontaneous coffee beans fermentation process.

Compounds	Fermentation assay (h)								
	0	6	12	18	24	30	36	42	48
**GC-MS** (**area** × **10**^**5**^)									
***Acids***									
1,2-Benzenedicarboxylic acid	5.51 ± 0.77^a^	2.85 ± 0.23^b^	2.77 ± 0.37^b^	2.47 ± 0.49^b^	5.11 ± 0.01^a^	5.11 ± 0.49^a^	5.93 ± 1.50^a^	2.47 ± 0.36^b^	1.86 ± 0.46^c^
2-Hydroxy-Benzoic acid	ND	4.18 ± 0.93^a^	3.88 ± 0.19^a^	2.89 ± 1.28^a^	2.67 ± 0.96^a^	2.46 ± 0.65^a^	2.61 ± 1.30^a^	2.27 ± 0.60^a^	2.69 ± 0.40^a^
Hexadecanoic acid	6.77 ± 2.95^a^	8.24 ± 0.60^a^	8.32 ± 2.83^a^	6.50 ± 1.79^a^	6.44 ± 1.52^a^	7.45 ± 2.89^a^	5.20 ± 0.75^a^	5.38 ± 1.03^a^	6.99 ± 1.10^a^
9,12-Octadecadienoic acid	11.9 ± 5.67^ab^	11.4 ± 0.91^ab^	15.0 ± 8.07^a^	5.02 ± 2.20^b^	7.39 ± 3.14^ab^	6.27 ± 1.32^ab^	3.09 ± 0.89^b^	8.03 ± 4.82^ab^	2.22 ± 0.21^b^
9,12,15-Octadecatrienoic acid	6.88 ± 3.53^ab^	6.65 ± 0.56^ab^	7.13 ± 3.92^a^	3.10 ± 1.36^abc^	3.31 ± 0.98^abc^	3.33 ± 0.85^abc^	2.26 ± 0.71^bc^	2.78 ± 0.55^abc^	1.41 ± 0.44^c^
***Alcohol***									
1-Hexanol	ND	ND	ND	3.61 ± 0.57^a^	4.80 ± 0.22^b^	4.26 ± 0.78^ab^	5.06 ± 0.14^b^	2.59 ± 0.43^c^	4.77 ± 0.35^c^
2-Heptanol	ND	ND	ND	1.93 ± 0.21^a^	3.38 ± 0.92^b^	3.76 ± 0.25^b^	3.30 ± 1.52^b^	2.70 ± 0.29^b^	2.46 ± 1.90^b^
2-Ethyl-1-hexanol	3.76 ± 0.09^ab^	2.24 ± 0.18^c^	3.44 ± 0.38^b^	3.34 ± 0.19^b^	3.62 ± 0.11^ab^	3.65 ± 0.47^ab^	4.32 ± 0.67^a^	3.61 ± 0.22^ab^	3.85 ± 0.40^ab^
*Aldehyde*									
Nonanal	ND	ND	ND	5.54 ± 1.54^ab^	4.51 ± 1.47^abc^	5.99 ± 1.45^b^	5.51 ± 0.84^ab^	3.25 ± 0.06^c^	3.72 ± 0.22^ac^
***Ester***									
Isoamyl acetate	ND	ND	ND	0.60 ± 0.14^a^	0.99 ± 0.28^bc^	0.86 ± 0.15^bc^	1.00 ± 0.01^c^	0.71 ± 0.10^ab^	0.58 ± 0.00^a^
2,2,6-Trimethyloctane	ND	ND	1.80 ± 0.45^a^	2.18 ± 0.18^b^	2.24 ± 0.05^c^	1.41 ± 0.03^c^	1.23 ± 0.02^a^	1.07 ± 0.08^a^	ND
1-Pentadecanal	13.0 ± 9.64^a^	2.12 ± 0.46^b^	1.35 ± 0.54^b^	3.9 ± 2.51^bc^	13.1 ± 3.93^a^	11.6 ± 3.78^ac^	13.0 ± 3.40^a^	1.33 ± 0.63^b^	1.94 ± 0.22^b^
Isopropyl palmitate	ND	4.47 ± 3.58^a^	2.01 ± 0.10^a^	ND	1.85 ± 0.99^a^	1.43 ± 0.02^a^	2.89 ± 0.34^a^	5.23 ± 4.32^a^	1.78 ± 0.76^a^
***Ether***									
n-Octyl ether	5.23 ± 5.83^a^	29.9 ± 28.3^b^	8.59 ± 2.84^ab^	2.02 ± 1.87^a^	5.15 ± 1.04^a^	4.39 ± 0.16^a^	6.25 ± 0.15^a^	7.36 ± 6.33^ab^	6.93 ± 3.91^ab^
9,12-Octandecadien-1-ol	4.31 ± 1.92^ab^	7.11 ± 1.55^a^	6.52 ± 2.01^ab^	3.21 ± 1.88^b^	5.05 ± 2.06^ab^	4.01 ± 0.26^ab^	ND	3.78 ± 1.28^ab^	ND
*Furan*									
2-Furanmethanol	2.28 ± 0.37^ab^	1.48 ± 0.09^a^	1.52 ± 0.16^a^	2.22 ± 0.36^ab^	2.71 ± 0.84^b^	1.90 ± 0.15^ab^	2.08 ± 0.83^ab^	2.02 ± 0.18^ab^	1.74 ± 0.21^ab^
***Hydrocarbon***									
2,3,6-Trimethyloctane	2.84 ± 0.96^ac^	1.63 ± 0.41^b^	1.87 ± 0.16^bc^	3.18 ± 0.02^a^	4.64 ± 0.31^d^	4.21 ± 0.15^d^	4.31 ± 0.12^d^	2.69 ± 0.24^ac^	2.99 ± 0.49^a^
5-Isobutylnonane	6.68 ± 1.04^a^	1.54 ± 1.30^b^	0.95 ± 0.69^b^	7.82 ± 0.24^a^	8.22 ± 2.08^a^	7.90 ± 1.52^a^	6.56 ± 2.19^a^	ND	ND
***Phenol***									
2,4-Di-tert-butylphenol	3.72 ± 2.38^a^	3.55 ± 0.70^a^	2.67 ± 0.12^ab^	2.76 ± 0.52^ab^	2.19 ± 1.07^ab^	1.17 ± 0.18^b^	ND	2.60 ± 0.37^ab^	3.25 ± 0.47^ab^
***Terpene***									
D-Limonene	1.51 ± 0.41^a^	2.57 ± 0.13^b^	2.73 ± 0.35^b^	1.23 ± 0.10^a^	1.31 ± 0.27^a^	1.42 ± 0.18^a^	1.38 ± 0.01^a^	3.20 ± 0.10^b^	2.45 ± 0.83^b^
Linalool	5.08 ± 1.35^a^	6.73 ± 0.56^ab^	5.43 ± 0.84^a^	8.05 ± 0.14^abc^	10.8 ± 0.23^bc^	9.28 ± 2.24^bc^	11.5 ± 3.5^c^	8.87 ± 0.76^abc^	7.61 ± 0.99^ab^

The chemical composition of fresh (unfermented) and fermented coffee beans was analysed by HPLC and GC-MS (Table 3). The Colombian coffee beans constitution had 7.28 g/L glucose, 5.26 g/L of fructose, 0.18 g/L ethanol and 2.76 g/L of organic acids content. The coffee bean composition remained unchanged after the fermentation and drying processes. This indicates that microbial activity and drying process do not interfere in the composition of major compounds inside the coffee beans. The major organic acids and sugars concentration in the fermented coffee beans is similar to those found in Brazil and Ecuador^10,36^, and are considered key precursors of coffee-impacting molecules generated during the roasting^5^.

**Table 3:** HPLC and GC-MS analysis of fresh (unfermented) and fermented coffee beans from Colombian process.

Compounds (retention time)	Aroma perception	Fresh coffee beans	Fermented coffee beans
**HPLC** (**g L**^**−1**^)			
Glucose	Sweetness	7.28 ± 1.36^a^	6.80 ± 0.41^a^
Fructose	Sweetness	5.26 ± 1.09^a^	5.09 ± 0.22^a^
Ethanol	Alcoholic	0.18 ± 0.01^a^	0.19 ± 0.01^a^
Citric Acid	Sour	2.54 ± 0.62^a^	2.26 ± 0.18^a^
Lactic Acid	Sour	0.16 ± 0.02^a^	0.20 ± 0.06^a^
Acetic Acid	Vinegar	0.03 ± 0.01^a^	0.08 ± 0.01^b^
Succinic Acid	Bitter	0.03 ± 0.00^a^	0.03 ± 0.00^a^
**GC-MS** (Area *10^**5**^)			
***Acids***			
3-Methylbutanoic acid (4.0)	Sweaty, acidic	16.85 ± 1.9^a^	20.70 ± 4.5^a^
2-Methylbutanoic acid (4.2)	Sweaty, acidic	4.99 ± 1.6^a^	5.21 ± 0.9^a^
***Alcohols***			
1-Octen-3-ol (9.7)	Green, oily, vegetative	2.27 ± 1.1^a^	6.94 ± 2.14^a^
***Aldehydes***			
Acetaldehyde (1.8)	Fruity	0.06 ± 0.02^a^	0.55 ± 0.04^b^
Nonanal (16.9)	Citrus	3.75 ± 0.4^a^	5.29 ± 0.4^b^
2-Heptenal (8.3)	Green, fatty	1.3 ± 1.0^a^	6.7 ± 2.31^b^
Benzaldehyde (8.5)	Fruity, cherry	15.08 ± 1.64^a^	37.95 ± 5.19^b^
n-Caprylaldehyde (11.3)	Fruity	1.91 ± 0.0^a^	2.71 ± 0.6^a^
Benzeneacetaldehyde (13.6)	Honey, floral	33.45 ± 5.74^a^	72.13 ± 16.82^b^
***Alkanes***			
3,3,5-Trimethylheptane (12.4)	NF	1.53 ± 0.51^a^	1.95 ± 0.38^a^
5-Isobutylnonane (14.5)	NF	10.73 ± 0.48^a^	13.08 ± 4.16^a^
Tetradecane (21.1)	Waxy	2.53 ± 0.37^a^	2.98 ± 0.72^a^
3-Methyl-5-propylnonane (22.4)	NF	2.07 ± 0.95^a^	2.59 ± 1.92^a^
4,6-Dimethyldodecane (23.3)	Floral	11.60 ± 5.49^a^	13.43 ± 9.63^a^
Heneicosane (24.3)	Waxy	2.01 ± 1.27^a^	2.30 ± 1.70^a^
***Terpenes***			
D-Limonene (12.7)	Sweet, citrus	2.96 ± 1.32^a^	2.48 ± 0.54^a^
***Aromatics***			
Styrene (5.5)	Sweet, floral	4.78 ± 0.83^a^	10.00 ± 5.27^a^

HPLC.: high performance liquid chromatography; GC-MS.: gas chromatography–mass spectrometry; NF.: Not found.

A total of 15 volatiles were detected by GC-MS in fresh (unfermented) and fermented coffee beans, including organic acids, alcohols, aldehydes, alkanes, terpenes, and aromatics (Table 3). 3-Methylbutanoic acid, benzaldehyde, benzeneacetaldehyde, 5-isobutylnonane, and 4,6-dimethyldodecane were the compounds found in high concentrations in both fresh and fermented coffee beans. Interestingly, most of these compounds have increased significantly after the fermentation process. In particular, the increase of aldehydes (*e*.*g*., acetaldehyde, benzaldehyde, benzeneacetaldehyde, nonanal, octanal) and 1-octen-3-ol content can be attributed to the domination of *P*. *nakasei*, *Candida* sp., *Leuconostoc*, *Lactobacillus*, and *Lactococcus* (Figs 1 and 2). As the volatile compounds present low olfactory threshold, their incorporation in the chemical composition of the beans can attribute desirable and pleasant floral, fruity and citric sensory notes to the final beverage. On the other hand, some compounds, such as styrene and limonene, may have been originated from the seed germination during the processing^3,59^.

In summary, the Colombian spontaneous coffee beans fermentation process was characterised by the dominance of *Leuconostoc* and *Pichia nakasei*. The metabolic activity of these microbial groups resulted mainly in the production of lactic acid and acetaldehyde. In addition, 170 other microbial groups were present, contributing to the formation of a complex array of metabolites. Interestingly, 56 fungal and bacterial genera were reported for the first time in the coffee fermentation process. These microorganisms are mainly associated with local environments and migration from proximal biomes, indicating the formation of microbial niche-specific and metabolic activity in the Colombian spontaneous coffee fermentation process. Our data contribute to a better understanding of microbiome composition and open perspectives on their application in the enhancement of coffee fermentation process, such as production of high-quality coffee beans, selection of specific microbial groups for flavour modulation, and potential candidates for biological markers of Colombian coffees.

## Methods

### Fermentation

Coffee beans fermentation samples were collected from a coffee farm located in Buesaco, Nariño, Colombia (1°23′05″N, 77°09′23″W). The coffee farm is at 1,959 m altitude and produces ‘washed coffee’ according to the traditional method of fermentation practiced in Colombia. 10 kg of freshly harvested coffee cherries (*Coffea arabica* L.) were mechanically pulped and placed in cement tanks. Approximately, 4 L of water was added and a natural fermentation was allowed to occur by 48 h. Samples of 10 mL of the liquid fraction of fermenting coffee-bean mass at 0, 6, 12, 18, 24, 36, and 48 h were collected in triplicate. The collected samples were mixed and frozen in sterilized Falcon tubes at −20 °C for the further analysis.

### Total DNA extraction and high-throughput sequencing

Samples of 1 mL of the liquid fraction of fermenting coffee-bean mass at 0, 6, 12, 18, 24, 36, 48 h were used for total DNA extraction. The samples were centrifuged (12,000 × g, 1 min) and the supernatant removed. The pellets were suspended in 500 µL of Tris-EDTA, homogenized with 10 µL of lysozyme solution (Sigma-Aldrich, Arklow, Ireland), and incubated at 30 °C for 60 min. Then, 50 µL of SDS 10% (w/v) and 10 μL of proteinase K (Sigma-Aldrich) were added, followed by homogenization and incubation at 60 °C for 60 min. A volume of 150 µL of phenol/chloroform (25:24; Sigma-Aldrich) was added, homogenized by inversion and centrifuged (12,000 × g, 5 min). Finally, supernatant was removed and the DNA precipitated with absolute ethanol (Sigma-Aldrich). Pellets were washed with 80% ethanol, dried and suspended in Mili-Q^®^ ultrapure water (Merck, Darmstadt, Germany). Extracted DNA quality was cheked on a 0.8% (w/v) agarose gel and quantified with the Nanodrop 2000 spectrophotometer (Thermo Fisher Scientific, Waltham, MA, USA). Twenty ng of the extracted DNA, containing complementary adaptors for Illumina platform^60^, were amplified using degenerated primers for the hypervariable V4 region of both 16S (515F and 806R) and 18S (5287F and 706R) rRNA genes. PCR conditions for the generation of bar-coded amplicons are as follow: initial denaturation at 95 °C for 3 min; followed by 18 cycles of denaturation at 95 °C for 30 s; annealing at 50 °C for; extension at 68 °C for 60 s, and a final extension at 68 °C for 10 min. The PCR products were quantified using the Qubit dsDNA HS kit (Invitrogen, Carlsbad, CA, USA). Raw sequences obtained were analysed using the standard parameters of QIIME (Quantitative Insights into Microbial Ecology) software, version 1.9.0^61^. Low quality reads, short sequences (<100 bp), and sequences containing more than one ambiguous base (N) were removed. Then, the high quality reads obtained were aligned against the SILVA database^62^ using the UCLUST method^63^. The taxonomic allocation and generation of the operational taxonomic units (OTUs) were performed using a 97% sequencing identity as cut-off. Shanghai, China. ACE, Chao 1, Shannon, and Simpson index were used to analyze the richness and biodiversity of the microbial communities.

### Major compounds quantified by High Performance Liquid Chromatography (HPLC) and Gas Chromatography (GC)

At every 6 h, the concentration of sugars (glucose and fructose), organic acids (citric, lactic, acetic and succinic acids), and ethanol of the liquid fraction of fermenting coffee beans was determined using high-performance liquid chromatography (HPLC), according to Carvalho Neto *et al*.^47^ Samples (1 mL) of the fermenting coffee pulp-bean mass (liquid fraction) were centrifuged at 6,000 × g (centrifuge model A14; Jouan SA, Saint-Herblain, France). Then, the supernatant was diluted at a 1:5 ratio with distilled water and filtered using a 0.22-µm pore size filter (Filtrilo, Colombo, Brazil). Filtered samples were injected into HPLC system equipped with a Hi‐Plex H column (300 × 7.7 mm; Agilent, Santa Clara, USA) and a refractive index (RI) detector (model HPG1362A; Hewlett-Packard Company, São Paulo, Brazil). The column was eluted in isocratic mode with a mobile phase of 5 mM H_2_SO_4_ at 60 °C and a flow rate of 0.6 mL/min. Major volatile compounds were quantified by gas chromatography (GC). Samples of 5 mL of the fermenting coffee pulp-bean mass (liquid fraction) plus NaCl 5% (w/v) were disposed in hermetic sealed vials (20 mL). In order to achieve a headspace equilibrium, samples were heated (65 °C) and agitated (150 rpm) on a hot plate with a magnetic stirrer (IKA®, Campinas, Brazil) for 10 min. The volatiles present in the headspace were manually extracted with a 1.0 mL syringe (Hamilton^®^, Reno, NV, USA) and injected into a gas chromatographer (GC) (Shimadzu model 17 A, Tokyo, Japan) equipped with a HP‐5 capillary column (30 m × 0.32 mm × 0.25 µm). The working conditions within the GC were: column at a range 40–150 °C (rate of 20 °C/min), injector at 250 °C, and detector at 250 °C. Nitrogen was the carrier gas used, at a flow rate of 1.5 mL/min, column press of 50 kPa, and split ratio of 1:5. The volatile compounds were identified comparing the peak retention times against standards. For quantification, standard solutions of ethanol were prepared in concentration levels (1, 10, 20, 50, 100 and 1000 μmol/L) and used to construct calibration curves. The quantification of volatile compounds was expressed as ethanol equivalent.

### Minor compounds identified by gas chromatographer coupled to a mass spectrophotometry

The volatile compounds from fermenting coffee pulp-bean mass (liquid fraction) were analysed by Solid Phase Microextraction (SPME), using a DVB/CAR/PDMS Fibre (Supelco Co., Bellefonte, PA USA) and injected into gas chromatographer coupled to a mass spectrophotometry connected to an autosampler (GCMS2010 Plus, TQ8040, AO 5000; Shimadzu, Tokyo, Japan). The sample was prepared by diluting two milliliters at 1:1 ratio with distilled water plus NaCl 5% (w/v) and disposed in hermetic sealed vials (20 mL). The SPME fibre was exposed for 30 min at 60 °C. The compounds were thermally desorbed at 260 °C and directly introduced into the gas chromatograph. The GC was equipped with a capillary column (model SH-Rtx-5MS; 30 m × 0.25 mm × 0.25 µm). The temperature within the GC was at follows: column oven at 60 °C, injection at 260 °C, and detector at 250 °C. Helium was the carrier gas used, at a flow rate of 1 mL/min, column press of 57.4 kPa, and split ratio of 1:20. The mass spectrophotometry range was 30–250 (m/z), at an ion source temperature of 250 °C. Volatiles were identified comparing each mass spectrum either with the spectra from authentic compounds or with spectra in reference libraries. The relative abundance of each volatile compound present in the headspace was showed as peak area times 10^5^.

### Chemical composition of fermented coffee beans

After the end of the fermentation, coffee beans were dried at 45 °C during 72 h in a drying oven with air circulation (Thoth, Piracicaba, Brazil) until a humidity of 11% was reached. Then, beans were grounded using an electric coffee grinder. Dried, ground coffee beans were analysed by HPLC and GS-MS according to the procedures described above. Unfermented coffee beans were included as a control. For HPLC analysis, a cold extraction was done by adding 0.2 g of ground beans to 1 mL of distilled water. Samples were centrifuged, filtered using a 0.22-µm pore size filter (Filtrilo, Colombo, Brazil), and injected into the HPLC system.

### Statistical analysis

Statistical significance was calculated using post-hoc comparison of means using Duncan’s test. Analyses were performed using the SAS programme, version 7.0 (Statistical Analysis System, Cary, NC, USA). Level of significance was established using a two-sided p-value < 0.05.

## Supplementary information

Alpha rarefaction curves and relative abundance at the genus level from Colombian spontaneous coffee fermentation process
