# Supplementary material for: First description of bacterial and fungal communities in Colombian coffee beans fermentation analysed using Illumina-based amplicon sequencing
Source: Sci Rep. 2019 Jun 19;9:8794. doi: 10.1038/s41598-019-45002-8 (PMC6584692; doi:10.1038/s41598-019-45002-8)
Supplement: Supplementary file 1 — Alpha rarefaction curves and relative abundance at the genus level from Colombian spontaneous coffee fermentation process [file 41598_2019_45002_MOESM1_ESM.pdf]

SUPPLEMENTARY MATERIAL

First description of bacterial and fungal communities in Colombian coffee beans fermentation analysed using Illumina-based amplicon sequencing

Ana C. de Oliveira Junqueira[a], Gilberto V. de Melo Pereira[a], Jesus D. Coral Medina[b], María C. R. Alvear[b], Rubens Rosero[b], Dão P. de Carvalho Neto[a], Hugo G. Enríquez[b], and Carlos R. Soccol[a]*

[a] Bioprocess Engineering and Biotechnology Department, Federal University of Paraná (UFPR), 19011 Curitiba, Paraná, 81531-980, Brazil

[b] Process Engineering, Mariana University, Pasto, Nariño, Colombia

*Author for correspondence: Carlos Ricardo Soccol

E-mail adresses: soccol@ufpr.br

Tel.: +55 41 33 613 191;

Fax: +55 41 33 613 695

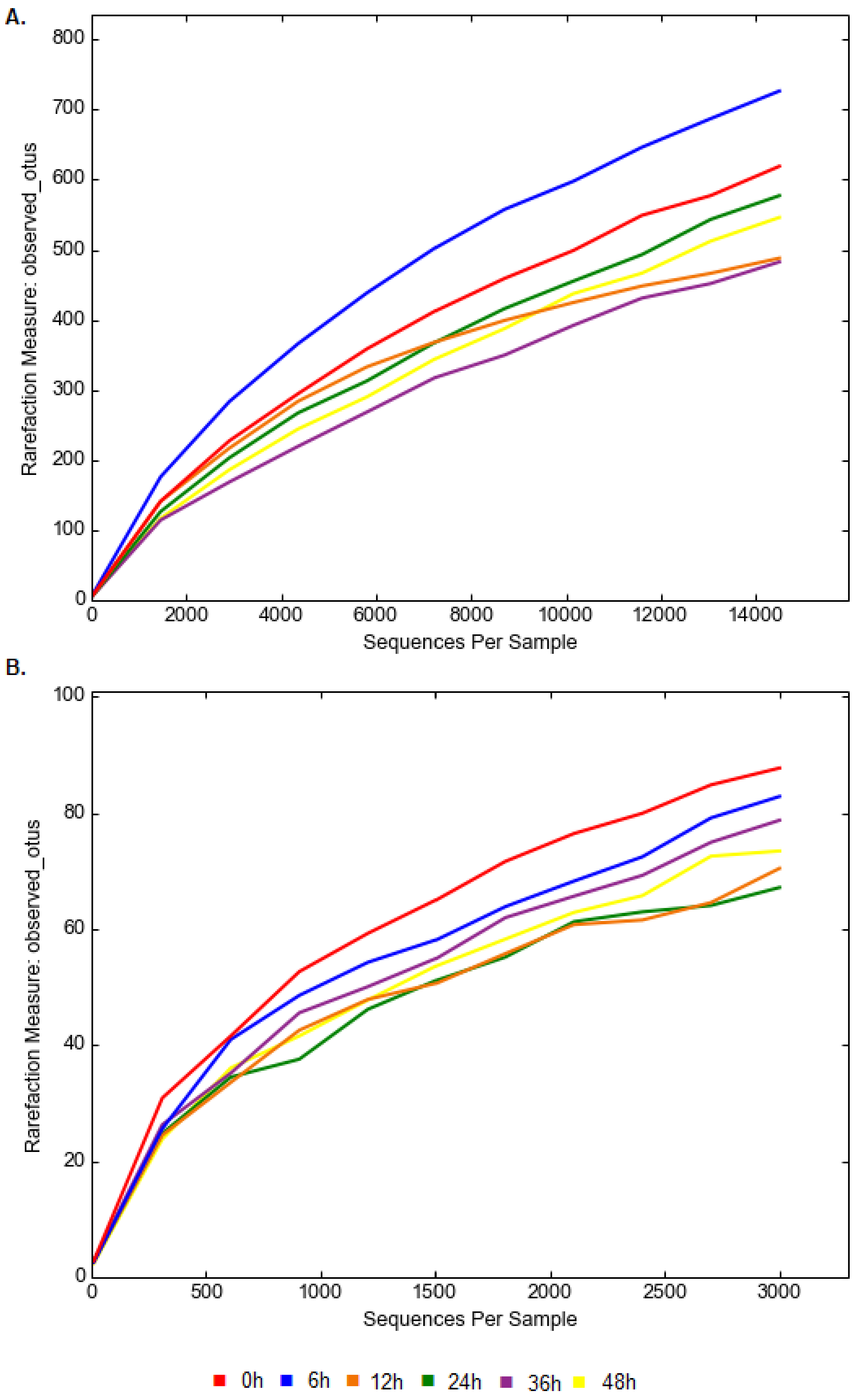

Fig. S1. Alpha rarefaction curves of observed OTUs (operational taxonomic units) from the temporal samples. **A.** Bacterial analysis, **B.** Fungal analysis.

Table S1. Relative abundance (%) of bacteria and fungi at the genus level during Colombian spontaneous coffee fermentation process. Bacteria reported for the first time in coffee fermentation are highlighted in red asterisk (*)

Family	Genera	Fermentation time (h)					
		0	6	12	24	36	48
Bacteria							
Leuconostocaceae	*Leuconostoc*	46.40	72.24	73.93	84.66	83.72	65.81
	Fructobacillus	0.08	-	0.10	0.09	0.29	0.40
	Oenococcus	-	-	-	-	-	0.03
	Weisella	0.02	-	-	0.09	1.14	1.17
Streptococcaceae	*Lactococcus*	20.38	0.41	1.81	5.22	1.97	1.21
Lactobacillaceae	*Lactobacillus*	0.17	0.84	1.03	0.95	7.53	27.28
	Pediococcus	0.01	-	0.02	0.02	0.03	0.07
Enterobacteriaceae	*Erwinia*	17.00	7.33	8.69	3.41	2.55	1.66
	Klebsiella	0.50	0.31	0.31	0.17	0.12	0.05
	Citrobacter	0.07	0.02	-	0.01	0.01	0.01
	Enterobacter	0.06	0.06	-	0.01	0.01	-
	Serratia	0.06	0.04	-	0.01	0.01	0.01
	*Plesiomonas**	0.03	-	-	0.01	0.01	-
	*Trabulsiella**	0.01	-	-	0.01	-	-
Acetobacteraceae	*Acetobacter*	0.19	0.37	0.31	0.05	0.08	0.06
	Gluconacetobacter	0.05	0.10	-	0.01	0.02	0.01
	Roseomonas	0.09	0.12	0.08	0.03	0.01	0.01
	Roseococcus	0.02	-	0.02	-	-	-
	Gluconobacter	3.03	3.30	1.43	0.80	0.48	0.35
Methylobacteriaceae	*Methylobacterium*	1.28	1.19	0.77	0.55	0.21	0.15
Sphingomonadaceae	*Sphingomonas*	0.67	0.80	0.49	0.37	0.14	0.15
	Sphingobium	0.14	0.33	0.18	0.11	0.02	0.03
	Novosphingobium	0.12	0.21	0.11	0.03	0.01	0.02
	Kaisobacter	0.04	0.10	0.14	0.05	0.02	0.01
	Sphingopyxis	-	-	-	0.01	-	-
Cytophagaceae	*Spirosoma*	0.46	0.55	0.20	0.12	0.10	0.08

	*Dyadobacter**	0.26	0.37	0.23	0.15	0.03	0.03
	Larkinella	0.07	0.02	0.09	0.01	0.01	0.01
	Hymenobacter	0.06	0.14	0.06	0.02	0.01	0.01
	*Rudanella**	0.01	0.02	0.02	-	-	-
	*Emticida**	-	0.08	-	-	-	-
	*Leadbetterella**	-	-	-	-	-	<0.01
Moraxellaceae	*Acinetobacter*	0.69	2.20	1.57	0.32	0.34	0.25
Sphingobacteriaceae	*Pedobacter*	0.55	0.49	0.25	0.19	0.06	0.04
	*Olivibacter**	0.09	-	-	-	-	-
	*Flavisolibacter**	0.05	-	0.05	0.01	-	-
	Sphingobacterium	0.04	0.08	0.10	-	0.01	0.01
	Sediminibacterium	0.04	-	-	-	-	-
	Niabella	0.02	-	-	-	-	-
	Segetibacter	-	-	0.05	-	-	-
Microbacteriaceae	*Pseudoclavibacter**	0.10	0.41	0.07	0.07	0.02	0.01
	Rathayibacter	0.04	0.12	0.11	0.01	0.01	0.01
	*Salinibacterium**	0.01	0.06	-	-	-	-
	*Cryocola**	0.01	0.02	-	-	-	-
Weeksellaceae	*Chryseobacterium*	0.54	0.16	0.13	0.03	0.05	0.07
	*Wautersiella**	-	0.06	0.26	-	-	<0.01
Pseudonocardiaceae	*Actinomycetospora*	0.12	0.19	0.06	0.02	-	0.01
	Pseudonocardia	0.23	0.21	-	0.04	0.01	0.01
Paenibacillaceae	*Saccharibacillus**	0.35	-	-	-	0.01	-
	Paenibacillus	-	-	0.02	<0.01	<0.01	<0.01
Comamonadaceae	*Polaromonas*	0.19	0.27	0.18	0.08	0.03	0.04
	Hylemonella	0.06	0.53	0.08	0.04	0.01	0.01
	Comamonas	0.03	0.04	0.02	0.01	0.01	0.01
	*Paucibacter**	-	-	-	-	<0.01	-
	*Rubrivivax**	0.01	-	-	0.02	<0.01	-
Caulobacteraceae	*Mycoplana*	0.19	0.33	0.03	0.08	0.03	0.01
	Atrhrospira	-	0.02	-	-	-	-
	Phenylobacterium	0.01	-	-	0.01	-	-

Xanthomonadaceae	*Stenotrophomonas*	0.13	0.27	0.22	0.05	0.05	0.03
	Luteimonas	0.05	0.10	0.09	0.09	0.01	0.01
	Luteibacter	0.02	0.10	0.05	0.03	0.01	0.01
Rhodobacteraceae	*Rubellimicrobium*	0.09	0.14	0.10	0.04	0.02	0.03
	Rhodobacter	0.09	0.10	-	0.02	-	0.01
Chitinophagaceae	*Flavisolibacter*	0.05	-	0.05	0.01	-	<0.01
	*Chitinophaga**	0.01	-	-	-	-	<0.01
	*Niabella**	0.02	-	-	-	-	-
	Sediminibacterium	0.04	-	-	-	<0.01	-
	Segetibacter	-	-	0.05	-	-	-
Rhizobiaceae	*Rhizobium*	0.09	0.06	0.08	0.02	0.01	0.01
	*Kaistia**	0.01	0.06	0.03	0.01	-	-
	Agrobacterium	1.09	1.05	0.34	0.51	0.10	0.09
Nocardiaceae	*Rhodococcus*	0.10	0.10	0.15	0.01	0.01	0.01
Hyphomicrobiaceae	*Rhodoplanes*	0.07	0.06	-	0.02	0.01	0.01
Beutenbergiaceae	*Salana**	0.06	0.12	-	0.02	-	-
Flavobacteriaceae	*Flavobacterium*	0.06	0.10	0.13	0.02	0.01	0.02
	*Capnocytophaga**	-	-	-	-	-	<0.01
Lachnospiraceae	*Ruminococcus*	0.03	-	0.10	0.03	0.01	0.02
	*Blautia**	-	0.04	0.04	0.01	-	0.01
	Coprococcus	-	-	-	-	<0.01	<0.01
	Dorea	0.01	-	-	<0.01	<0.01	<0.01
Gemmataceae	*Gemmata*	0.03	0.18	0.02	0.02	0.03	<0.01
Dermabacteraceae	*Brachybacterium*	-	0.02	0.12	-	0.04	0.03
Fimbriimonadaceae	*Fimbriimonas*	0.08	0.10	0.04	0.03	0.01	-
Micrococcaceae	*Arthrobacter**	0.01	-	-	-	0.01	-
	*Kocuria**	0.01	0.12	-	-	-	-
	*Micrococcus**	-	-	0.02	-	-	-
	*Rothia**	0.02	-	-	-	-	-
Kineosporiaceae	*Kineococcus*	0.04	0.10	0.02	0.02	0.01	0.02
	*Kineosporia**	0.02	-	0.04	0.02	0.01	0.01

Brevibacteriaceae	*Brevibacterium*	0.05	-	-	0.01	0.01	0.04
Bdellovibrionaceae	*Bdellovibrio*	0.04	0.04	0.02	0.01	-	-
Mycobacteriaceae	*Mycobacterium*	0.04	-	0.05	0.02	-	-
Nocardioidaceae	*Aeromicrobium*	0.01	-	-	0.02	-	0.01
	Nocardioides	0.03	-	-	0.02	0.01	-
Oxalobacteraceae	*Janthinobacterium*	0.03	0.08	0.04	0.01	0.01	0.01
	*Polynucleobacter**	-	-	0.02	-	-	-
Beijerinckiaceae	*Beijerinckia*	0.06	-	-	0.01	<0.01	-
Micromonsporaceae	*Actinoplanes*	0.03	-	0.07	0.01	-	<0.01
Porphyromonadaceae	*Dysgonomonas**	0.02	-	-	-	-	<0.01
	*Paludibacter**	-	-	-	-	-	<0.01
	*Parabacteroides**	0.02	-	-	-	-	-
Cellulomonadaceae	*Cellulomonas*	0.03	-	0.09	<0.01	-	0.01
Intrasporangiaceae	*Arseniococcus*	-	-	-	-	-	<0.01
	Terracoccus	0.03	-	-	-	<0.01	<0.01
Enterococcaceae	*Enterococcus*	0.03	-	-	0.01	<0.01	0.01
Phyllobacteriaceae	*Methylophilla**	-	-	-	0.02	-	<0.01
	*Methylosinus**	0.02	-	0.05	-	<0.01	-
	Aminobacter	0.03	0.06	0.03	0.01	<0.01	<0.01
Burkhoolderiaceae	*Burkholderia*	0.02	-	0.02	0.01	-	<0.01
	*Lautropia**	0.01	-	-	-	-	<0.01
Blattabacteriaceae	*Blattabacterium**	0.02	-	0.03	-	0.01	<0.01
Bacillaceae	*Bacillus*	0.02	-	-	-	<0.01	-
Ruminococcaceae	*Faecalibacterium*	0.01	0.02	-	0.01	0.01	0.02
	Oscillospira	0.01	-	-	0.01	<0.01	0.01
Brucellaceae	*Ochrobactrum*	0.02	-	0.02	0.01	0.01	0.01
Methylocystaceae	*Methylopila**	-	-	-	-	-	<0.01
	*Methylosinus**	0.02	-	0.05	0.03	<0.01	<0.01
Rhodospirillaceae	*Azospirillum*	-	-	0.02	-	-	-

	*Rhodospirillum**	0.02	-	-	-	-	-
Alcaligenaceae	*Achronobacter*	-	-	-	-	-	<0.01
	*Pigmentiphaga**	0.02	-	-	<0.01	<0.01	-
Chthoniobacteraceae	*Candidatus* Xiphinematobacter	0.02	-	0.02	-	-	-
	Chthoniobacter	-	-	0.03	-	-	-
Trueperaceae	*Truepera**	0.02	-	-	-	-	-
Actinomycetaceae	*Actinomyces*	0.01	-	-	<0.01	<0.01	<0.01
Clostridiaceae	*Clostridium*	0.01	-	-	<0.01	<0.01	<0.01
Gemmatimonadaceae	*Gemmatimonas*	0.01	-	-	0.02	-	<0.01
Alteromonadaceae	*Cellvibrio**	0.01	-	-	<0.01	-	<0.01
Corynebacteriaceae	*Corynebacterium*	-	-	-	<0.01	<0.01	<0.01
Geodermatophilaceae	*Geodermatophilus*	-	-	-	0.01	0.01	<0.01
Streptomycetaceae	*Streptomyces*	-	-	-	0.01	<0.01	<0.01
Williamsiaceae	*Williamsia**	-	-	-	<0.01	-	<0.01
Bifidobacteriaceae	*Bifidobacterium*	-	-	0.03	-	-	-
Patulibacteraceae	*Patulibacter**	-	-	0.02	-	<0.01	<0.01
Bacteroidaceae	*Bacteroides*	-	-	0.02	<0.01	<0.01	0.01
Prevotellaceae	*Prevotella*	-	-	-	-	<0.01	<0.01
Turicibacteraceae	*Turicibacter*	-	-	0.02	-	-	-
Fusobacteriaceae	*Fusobacterium*	-	-	-	<0.01	<0.01	<0.01
Nitrospiraceae	*Nitrospira*	-	-	-	-	<0.01	<0.01
Planctomycetaceae	*Planctomyces*	-	-	0.02	0.01	-	-
Bradyrhizobiaceae	*Bosea*	-	-	0.03	-	-	-
Xanthobacteraceae	*Labrys**	-	-	-	0.01	<0.01	<0.01
Rickettsiaceae	*Wolbachia*	-	0.04	-	<0.01	<0.01	-
Neisseriaceae	*Eikenela**	-	-	-	<0.01	<0.01	-
	*Neisseria**	-	-	-	<0.01	-	0.01

Nannocystaceae	*Nannocystis*	-	-	0.05	0.01	<0.01	<0.01
Campylobacteraceae	*Arcobacter*	-	-	0.04	-	-	-
	Campylobacter	-	-	-	<0.01	-	<0.01
Cardiobacteriaceae	*Cardiobacterium**	-	-	-	<0.01	-	<0.01
Legionellaceae	*Legionella*	-	-	0.03	0.01	-	<0.01
Pasteurellaceae	*Aggregatibacter**	-	-	-	<0.01	<0.01	<0.01
Sinobacteraceae	*Steroidobacter**	-	-	-	-	-	<0.01
Deinococcaceae	*Deinococcus**	-	-	-	<0.01	<0.01	<0.01
Fungi							
Saccharomycetaceae	*Pichia nakasei**	59.70	70.89	80	72.02	73.21	82.52
	Candida sp.	20.90	27.85	18	24.31	20.57	13.16
	*Candida vanderwaltii**	-	-	-	-	-	0.94
Dipodascaceae	*Dipodascus tetrasporeus**	11.94	0.63	-	1.84	2.87	2.61
Classiculaceae	Classiculaceae	4.48	0.63	-	0.92	0.48	0.19
Malasseziaceae	*Malassezia* sp.*	1.49	-	-	-	-	0.38
Saccharomycopsidaceae	Saccharoycopsidaceae	1.49	-	-	-	-	-
Pichiaceae	*Martiniozyma asiática**	-	-	2	-	0.48	-
Konodaceae	*Bensingtonia**	-	-	-	0.46	1.44	-
Tremellaceae	Tremellaceae	-	-	-	0.46	-	-
Lobulomycetales	Lobulomycetales*	-	-	-	-	0.48	-
Physciceae	Physciceae	-	-	-	-	0.48	-
Debaryomycetaceae	*Schwanniomyces* sp.	-	-	-	-	-	0.19

Note: Asterisk (*) indicates bacterial and fungal genera reported for the first time in coffee fermentation.

Table S2. Biotechnological applications of some bacteria reported in this study.

Family	Genus	Potential biotechnological applications	Ref.
Streptococcaceae	*Lactococcus*	Probiotic, produces nisin, modulates aroma in cheese ripening.	[1,2]
Acetobacteraceae	*Acetobacter, Gluconoacetobacter, Gluconobacter, Roseomonas, Roseococcus*	Cellulose biosynthesis, vinegar production.	[3,4],
Pseudomonadaceae	*Pseudomonas*	Production of enzymes and biosurfactants.	[5]
Sphingomonadaceae	*Sphingomonas, Novosphingobium, Kaistobacter, Sphingopyxis*	Bioremediation of toxic and recalcitrant molecules, biosynthesis of silver nanoparticles (antimicrobial activity), produces gibberellins and IAA (promotes plant growth), gellan gum biosynthesis.	[6 6–9]
Moraxellaceae	*Acinetobacter*	Hydrocarbon degrading activity, production of bioemulsifier (emulsan), bioremediation of heavy metal-contaminated waters.	[10,11]
Methylobacteriaceae	*Methylobacterium*	Able to grow from one-carbon compounds, conversion of CO_2 to value-added chemicals, improve plant's tolerance on arsenic, fix nitrogen in plants, enhancement of flavor in strawberries.	[10,12–14]
Cytophagaceae	*Spirosoma, Dyadobacter, Larkinella, Hymenobacter, Emticicia, Rudanella*	Bioremediation of contaminated sites by hydrocarbon.	[15,16]
Rhizobiaceae	*Agrobacterium,Rhizobium, Kaistia*	Inhabit the rhizosphere of plants, applied to recombinant DNA technology and plant biotechnology.	[17]
Comamonadaceae	*Polaromonas, Paucibacter Hylemonella*	Ability to degrade naphthalene and cyclic cyanobacterial hepatoxins.	[18,19]
Microbacteriaceae	*Agrococcus, Cryocola, Curtobacterium, Mycetocola, Frigoribacterium, Salinibacterium, Rathayibacter*	Stains are isolated from a range of environments (from wall painting, permafrost soil, surface of cheese, and desert), but further studies regard its applications are still required.	[20–23]
Xanthomonadaceae	*Stenotrophomonas, Luteimonas, Luteibacter, Dokdonella*	Promotes plant-growth, antifungal activity, production of enzymes (cold-active xylanase, lipase), bioremediation of dye effluent.	[24–29]
Sphingobacteriaceae	*Pedobacter, Olivibacter, Sphingobacterium*	Biodegradation of a range of molecules, such as, poly(aspartic acid), hydrocarbon, 17α-ethynylestradiol.	[30–32]
Chitinophagaceae	*Flavisolibacter, Sediminibacterium, Niabella, Chitinophaga*	Antibiotics production, biosynthesis of aroma compounds.	[33–35]

Actinomycetales	*Actinomycetaceae, Beutenbergiaceae, Brevibacteriaceae, Cellulomonadaceae, Corynebacteriaceae, Dermabacteraceae, Frankiaceae, Geodermatophilaceae, Intrasporangiaceae, Kineosporiaceae, Micrococcaceae, Micromonosporaceae, Mycobacteriaceae, Nakamurellaceae, Nocardiaceae, Nocardioidaceae, Pseudonocardiaceae, Streptomycetaceae, Williamsiaceae*	Production of a wide range of antibiotics, pigments, amino acids, enzymes, and organic acids. Some strain are radiation resistant. Bioremediation of oil- and metal-contaminated sites due the ability to decompose a great variety of molecules.	[36–53]
Aeromonadales	*Aeromonadaceae*	Ability to degrade lignin.	[54]
Alteromonadales	*Alteromonadaceae*	Production of exopolysaccharides with potential applications.	[55]
Armatimonadales	*Armatimonadaceae*		
Bacillales	*Bacillaceae, Paenibacillaceae*	Biological control against pathogen in plants rhizosphere.	[56]
Bacteroidales	*Bacteroidaceae, Porphyromonadaceae, Prevotellaceae, Rikenellaceae*	Gastrointestinal tract microbiome with great potential as new-generation of probiotics.	[57]
Bdellovibrionales	*Bacteriovoracaceae, Bdellovibrionaceae*	Wastewater treatment.	[58]
Bifidobacteriales	*Bifidobacteriaceae*	Probiotic.	[59]
Burkholderiales	*Alcaligenaceae, Burkholderiaceae, Oxalobacteraceae*	Treatment of textile dyeing wastewater. Production of violacein (dark-violet pigment) with anticancer property.	[60,61]
Caulobacterales	*Caulobacteraceae*	Degradation of linear alkybenzene sulfonate.	[62]
Chthoniobacterales	*Chthoniobacteraceae*		
Clostridiales	*Christensenellaceae, Clostridiaceae, Ruminococcaceae, Lachnospiraceae*	Digestive tract-associated microbiome.	[63]
Deinococcales	*Deinococcaceae*	Synthesis and extracellular accumulation of silver nanoparticles.	[64]
Erysipelotrichales	*Erysipelotrichaceae*		
Fimbriimonadales	*Fimbriimonadaceae*		
Flavobacteriales	*Blattabacteriaceae, Flavobacteriaceae, Weeksellaceae*	Production of antioxidant pigment.	[65]
Fusobacteriales	*Fusobacteriaceae*		

Gemmatales	*Gemmataceae, Isosphaeraceae*		
Gemmatimonadales	*Gemmatimonadaceae*	Biosynthesis of carotenoid (oscillol 2,2'-dirhamnoside).	66
Lactobacillales	*Aerococcaceae, Enterococcaceae*	Probiotic and starter culture.	67,68
Legionellales	*Legionellaceae*		
Myxococcales	*Cystobacterineae, Haliangiaceae, Nannocystaceae, Polyangiaceae*	Biosynthesis of pharmaceutical relevant anticancer and antifungal metabolites.	69–71
Neisseriales	*Neisseriaceae*		
Nitrospirales	*Nitrospiraceae*	Nitrite and iron oxidizers. Degradation of organic compounds.	72
Pasteurellales	*Pasteurellaceae*	Potential pathogens and/or specialized commensals.	73
Pirellulales	*Pirellulaceae*		
Rhizobiales	*Beijerinckiaceae, Brucellaceae, Phyllobacteriaceae, Xanthobacteraceae, Methylocystaceae, Bradyrhizobiaceae, Hyphomicrobiaceae*	Microbiome associated to plants that have benefits such as nitrogen fixing, biosynthesis of nutrients and phytohormones.	74
Rhodobacterales	*Hyphomonadaceae, Rhodobacteraceae*	Microbial community present in coastal marine waters.	75
Rhodocyclales	*Rhodocyclaceae*	Able to degrade aromatic molecules.	76
Rhodospirillales	*Rhodospirillaceae*	Biosynthesis of biodegradable polyester.	77
Rickettsiales	*Rickettsiaceae*		
Saprospirales	*Saprospiraceae*	Hydrolysis and utilization of complex carbon sources.	78
Solirubrobacterales	*Conexibacteraceae, Patulibacteraceae, Solirubrobacteraceae*	Biodegradation of pharmaceutically active compounds (PhACs).	79
Verrucomicrobiales	*Verrucomicrobiaceae*	Hydrolytic enzyme activity.	80
Xanthomonadales	*Sinobacteraceae*	Rubber-degrading potential.	81

References

1. van Mastrigt, O., Gallegos Tejeda, D., Kristensen, M. N., Abee, T. & Smid, E. J. Aroma formation during cheese ripening is best resembled by Lactococcus lactis retentostat cultures. *Microb. Cell Fact.* **17,** 104 (2018).

2. Costas Malvido, M., Alonso González, E., Outeiriño, D. & Pérez Guerra, N. Production of a highly concentrated probiotic culture of Lactococcus lactis CECT 539 containing high amounts of nisin. *3 Biotech* **8,** 292 (2018).

3. Nguyen, V. T., Flanagan, B., Gidley, M. J. & Dykes, G. A. Characterization of Cellulose Production by a Gluconacetobacter xylinus Strain from Kombucha. *Curr. Microbiol.* **57,** 449–453 (2008).

4. Chauhan, P. S. & Jha, B. Pilot scale production of extracellular thermo-alkali stable laccase from Pseudomonas sp. S2 using agro waste and its application in organophosphorous pesticides degradation. *J. Chem. Technol. Biotechnol.* **93,** 1022–1030 (2018).

5. Silva, M. A. M. *et al.* Production of Biosurfactants by Pseudomonas Species for Application in the Petroleum Industry. *Water Environ. Res.* **89,** 117–126 (2017).

6. Wu, H. *et al.* Bioaugmentation potential of a newly isolated strain Sphingomonas sp. NJUST37 for the treatment of wastewater containing highly toxic and recalcitrant tricyclazole. *Bioresour. Technol.* **264,** 98–105 (2018).

7. Du, J., Singh, H. & Yi, T.-H. Biosynthesis of silver nanoparticles by Novosphingobium sp. THG-C3 and their antimicrobial potential. *Artif. Cells, Nanomedicine, Biotechnol.* **45,** 211–217 (2017).

8. Khan, A. L. *et al.* Bacterial endophyte Sphingomonas sp. LK11 produces gibberellins and IAA and promotes tomato plant growth. *J. Microbiol.* **52,** 689–695 (2014).

9. Sá-Correia, I. *et al.* Gellan gum biosynthesis in Sphingomonas paucimobilis ATCC 31461: Genes, enzymes and exopolysaccharide production engineering. *J. Ind. Microbiol. Biotechnol.* **29,** 170–176 (2002).

10. Nakar, D. & Gutnick, D. L. Involvement of a protein tyrosine kinase in production of the polymeric bioemulsifier emulsan from the oil-degrading strain Acinetobacter lwoffii RAG-1. *J. Bacteriol.* **185,** 1001–9 (2003).

11. Boswell, C. D., Dick, R. E. & Macaskie, L. E. The effect of heavy metals and other environmental conditions on the anaerobic phosphate metabolism of Acinetobacter johnsonii. *Microbiology* **145,** 1711–1720 (1999).

12. Sy, A. *et al.* Methylotrophic Methylobacterium bacteria nodulate and fix nitrogen in symbiosis with legumes. *J. Bacteriol.* **183,** 214–20 (2001).

13. Alcántara-Martínez, N., Figueroa-Martínez, F., Rivera-Cabrera, F., Gutiérrez-Sánchez, G. & Volke-Sepúlveda, T. An endophytic strain of Methylobacterium sp. increases arsenate tolerance in Acacia farnesiana (L.) Willd: A proteomic approach. *Sci. Total Environ.* **625,** 762–774 (2018).

14. Zabetakis, I. Enhancement of flavour biosynthesis from strawberry (Fragaria x ananassa) callus cultures by Methylobacterium species. *Plant Cell. Tissue Organ Cult.* **50,** 179–183 (1997).

15. Ali, N. *et al.* Autochthonous bioaugmentation with environmental samples rich in hydrocarbonoclastic bacteria for bench-scale bioremediation of oily seawater and desert soil. *Environ. Sci. Pollut. Res.* **23,** 8686–8698 (2016).

16. Willumsen, P. A., Johansen, J. E., Karlson, U. & Hansen, B. M. Isolation and taxonomic affiliation of N-heterocyclic aromatic hydrocarbon-transforming bacteria. *Appl. Microbiol. Biotechnol.* **67,** 420–428 (2005).

17. Hooykaas, P. J. J. *et al.* Agrobacterium-Mediated Transformation of Yeast and Fungi. in 1–26 (Springer, Berlin, Heidelberg, 2018). doi:10.1007/82_2018_90

18. Jeon, C. O., Park, W., Ghiorse, W. C. & Madsen, E. L. Polaromonas naphthalenivorans sp. nov., a naphthalene-degrading bacterium from naphthalene-contaminated sediment. *Int. J. Syst. Evol. Microbiol.* **54,** 93–97 (2004).

19. Rapala, J. *et al.* Paucibacter toxinivorans gen. nov., sp. nov., a bacterium that degrades cyclic cyanobacterial hepatotoxins microcystins and nodularin. *Int. J. Syst. Evol. Microbiol.* **55,** 1563–1568 (2005).

20. Luo, X. *et al.* Mycetocola manganoxydans sp. nov., an actinobacterium isolated from the Taklamakan desert. *Int. J. Syst. Evol. Microbiol.* **62,** 2967–2970 (2012).

21. Bora, N. *et al.* Mycetocola reblochoni sp. nov., isolated from the surface microbial flora of Reblochon cheese. *Int. J. Syst. Evol. Microbiol.* **58,** 2687–2693 (2008).

22. Hansen, A. A. *et al.* Viability, diversity and composition of the bacterial community in a high Arctic permafrost soil from Spitsbergen, Northern Norway. *Environ. Microbiol.* **9,** 2870–2884 (2007).

23. Wieser, M. *et al.* Agrococcus citreus sp. nov., isolated from a medieval wall painting of the chapel of Castle Herberstein (Austria). *Int. J. Syst. Bacteriol.* **49,** 1165–1170 (1999).

24. Han, Z., Shang-guan, F. & Yang, J. Characterization of a novel cold-active xylanase from Luteimonas species. *World J. Microbiol. Biotechnol.* **34,** 123 (2018).

25. Bresciani, F. R. *et al.* Production and activity of extracellular lipase from Luteibacter sp. *Ann. Microbiol.* **64,** 251–258 (2014).

26. Liu, Y., Jin, J.-H., Liu, H.-C. & Liu, Z.-P. Dokdonella immobilis sp. nov., isolated from a batch reactor for the treatment of triphenylmethane dye effluent. *Int. J. Syst. Evol. Microbiol.* **63,** 1557–1561 (2013).

27. Rojas-Solís, D. *et al.* Pseudomonas stutzeri E25 and Stenotrophomonas maltophilia CR71 endophytes produce antifungal volatile organic compounds and exhibit additive plant growth-promoting effects. *Biocatal. Agric. Biotechnol.* **13,** 46–52 (2018).

28. Berg, G., Fritze, A., Hagemann, M. & Wolf, A. Stenotrophomonas rhizophila sp. nov., a novel plant-associated bacterium with antifungal properties. *Int. J. Syst. Evol. Microbiol.* **52,** 1937–1944 (2002).

29. Schmidt, C. S., Alavi, M., Cardinale, M., Müller, H. & Berg, G. Stenotrophomonas rhizophila DSM14405T promotes plant growth probably by altering fungal communities in the rhizosphere. *Biol. Fertil. Soils* **48,** 947–960 (2012).

30. Tabata, K., Kasuya, K. I., Abe, H., Masuda, K. & Doi, Y. Poly(aspartic acid) degradation by a Sphingomonas sp. isolated from freshwater. *Appl. Environ. Microbiol.* **65,** 4268–70 (1999).

31. Szabo, I. *et al.* Olivibacter oleidegradans sp. nov., a hydrocarbon-degrading bacterium isolated from a biofilter clean-up facility on a hydrocarbon-contaminated site. *Int. J. Syst. Evol. Microbiol.* **61,** 2861–2865 (2011).

32. Haiyan, R., Shulan, J., ud din Ahmad, N., Dao, W. & Chengwu, C. Degradation characteristics and metabolic pathway of 17α-ethynylestradiol by Sphingobacterium sp. JCR5. *Chemosphere* **66,** 340–346 (2007).

33. Beckmann, A., Hüttel, S., Schmitt, V., Müller, R. & Stadler, M. Optimization of the biotechnological production of a novel class of anti-MRSA antibiotics from Chitinophaga sancti. *Microb. Cell Fact.* **16,** 143 (2017).

34. Jansen, R. *et al.* Elansolid A3, a Unique p-Quinone Methide Antibiotic from Chitinophaga sancti. *Chem. - A Eur. J.* **17,** 7739–7744 (2011).

35. Nawrath, T., Gerth, K., Müller, R. & Schulz, S. The Biosynthesis of the Aroma Volatile 2-Methyltetrahydrothiophen-3-one in the Bacterium Chitinophaga Fx7914. *ChemBioChem* **11,** 1914–1919 (2010).

36. Lee, J.-Y., Na, Y.-A., Kim, E., Lee, H.-S. & Kim, P. The Actinobacterium Corynebacterium glutamicum, an Industrial Workhorse. *J. Microbiol. Biotechnol.* **26,** 807–822 (2016).

37. von Wintzingerode, F. *et al.* Salana multivorans gen. nov., sp. nov., a novel actinobacterium isolated from an anaerobic bioreactor and capable of selenate reduction. *Int. J. Syst. Evol. Microbiol.* **51,** 1653–1661 (2001).

38. Nouioui, I. *et al.* High quality draft genome of Nakamurella lactea type strain, a rock actinobacterium, and emended description of Nakamurella lactea. *Stand. Genomic Sci.* **12,** 4 (2017).

39. Subashchandrabose, S. R. *et al.* Rhodococcus wratislaviensis strain 9: An efficient p-nitrophenol degrader with a great potential for bioremediation. *J. Hazard. Mater.* **347,** 176–183 (2018).

40. Kuddus, M. Cold-active enzymes in food biotechnology: An updated mini review. (2018). doi:10.7324/JABB.2018.60310

41. Bharagava, R. N. & Mishra, S. Hexavalent chromium reduction potential of Cellulosimicrobium sp. isolated from common effluent treatment plant of tannery industries. *Ecotoxicol. Environ. Saf.* **147,** 102–109 (2018).

42. Apinya, T., Sombatsompop, N. & Prapagdee, B. Selection of a Pseudonocardia sp. RM423 that accelerates the biodegradation of poly(lactic) acid in submerged cultures and in soil microcosms. *Int. Biodeterior. Biodegradation* **99,** 23–30 (2015).

43. de Lima Procópio, R. E., da Silva, I. R., Martins, M. K., de Azevedo, J. L. & de Araújo, J. M. Antibiotics produced by Streptomyces. *Brazilian J. Infect. Dis.* **16,** 466–471 (2012).

44. Yassin, A. F. *et al.* Williamsia serinedens sp. nov., isolated from an oil-contaminated soil. *Int. J. Syst. Evol. Microbiol.* **57,** 558–561 (2007).

45. Zhang, S. *et al.* Salt-tolerant and plant-growth-promoting bacteria isolated from high-yield paddy soil. *Can. J. Microbiol.* 1–11 (2018). doi:10.1139/cjm-2017-0571

46. Mounier, J. *et al.* Surface microflora of four smear-ripened cheeses. *Appl. Environ. Microbiol.* **71,** 6489–500 (2005).

47. Stackebrandt, E. & Schumann, P. Cellulomonadaceae. in *Bergey's Manual of Systematics of Archaea and Bacteria* 1–3 (John Wiley & Sons, Ltd, 2015). doi:10.1002/9781118960608.fbm00029

48. Gontia, I., Kavita, K., Schmid, M., Hartmann, A. & Jha, B. Brachybacterium saurashtrense sp. nov., a halotolerant root-associated bacterium with plant growth-promoting potential. *Int. J. Syst. Evol. Microbiol.* **61,** 2799–2804 (2011).

49. Hezbri, K. *et al.* Geodermatophilus pulveris sp. nov., a gamma-radiation-resistant actinobacterium isolated from the Sahara desert. *Int. J. Syst. Evol. Microbiol.* **66,** 3828–

3834 (2016).

50. Zhang, W., Niu, C., Fu, R.-Y. & Peng, Z.-Y. Mycobacterium tuberculosis H37Rv infection regulates alternative splicing in Macrophages. *Bioengineered* **9,** 203–208 (2018).

51. Liu, M. *et al.* Kineococcus xinjiangensis sp. nov., isolated from desert sand. *Int. J. Syst. Evol. Microbiol.* **59,** 1090–1093 (2009).

52. Uzair, B. *et al.* Isolation, purification, structural elucidation and antimicrobial activities of kocumarin, a novel antibiotic isolated from actinobacterium Kocuria marina CMG S2 associated with the brown seaweed Pelvetia canaliculata. *Microbiol. Res.* **206,** 186–197 (2018).

53. Golińska, P. *et al.* Synthesis of silver nanoparticles from two acidophilic strains of *Pilimelia columellifera* subsp. *pallida* and their antibacterial activities. *J. Basic Microbiol.* **56,** 541–556 (2016).

54. Billings, A. F. *et al.* Genome sequence and description of the anaerobic lignin-degrading bacterium Tolumonas lignolytica sp. nov. *Stand. Genomic Sci.* **10,** 106 (2015).

55. Mata, J. A. *et al.* Characterization of exopolysaccharides produced by three moderately halophilic bacteria belonging to the family Alteromonadaceae. *J. Appl. Microbiol.* **105,** 521–528 (2008).

56. Gómez-Lama Cabanás, C. *et al.* Bacillales Members from the Olive Rhizosphere Are Effective Biological Control Agents against the Defoliating Pathotype of Verticillium dahliae. *Agriculture* **8,** 90 (2018).

57. Tan, H., Zhao, J., Zhang, H., Zhai, Q. & Chen, W. Isolation of Low-Abundant Bacteroidales in the Human Intestine and the Analysis of Their Differential Utilization Based on Plant-Derived Polysaccharides. *Front. Microbiol.* **9,** 1319 (2018).

58. Özkan, M. *et al.* Application of Bdellovibrio bacteriovorus for reducing fouling of membranes used for wastewater treatment. *Turkish J. Biochem.* (2018).

59. Quigley, E. M. M. Bifidobacteria as Probiotic Organisms: An Introduction. *Microbiota Gastrointest. Pathophysiol.* 125–126 (2017). doi:10.1016/B978-0-12-804024-9.00012-4

60. Huang, J. *et al.* Microbial biodegradation of aniline at low concentrations by Pigmentiphaga daeguensis isolated from textile dyeing sludge. *Int. Biodeterior. Biodegradation* **129,** 117–122 (2018).

61. Masuelli, L. *et al.* Violacein, an indole-derived purple-colored natural pigment produced by Janthinobacterium lividum, inhibits the growth of head and neck carcinoma cell lines both in vitro and in vivo. *Tumor Biol.* **37,** 3705–3717 (2016).

62. Cortés-Lorenzo, C. *et al.* Two novel strains within the family Caulobacteraceae capable of degradation of linear alkylbenzene sulfonates as pure cultures. *Int. Biodeterior. Biodegradation* **85,** 62–65 (2013).

63. Meehan, C. J. & Beiko, R. G. A Phylogenomic View of Ecological Specialization in the Lachnospiraceae, a Family of Digestive Tract-Associated Bacteria. *Genome Biol. Evol.* **6,** 703–713 (2014).

64. Deobagkar, D., Kulkarni, R., Shaiwale, N. & Deobagkar, D. Synthesis and extracellular accumulation of silver nanoparticles by employing radiation-resistant Deinococcus radiodurans, their characterization, and determination of bioactivity. *Int. J. Nanomedicine* **10,** 963 (2015).

65. Jiménez, M. E. P., Pinilla, C. M. B., Rodrigues, E. & Brandelli, A. Extraction and partial

characterisation of antioxidant pigment produced by *Chryseobacterium* sp. kr6. *Nat. Prod. Res.* 1–9 (2018). doi:10.1080/14786419.2017.1423304

66. Takaichi, S., Maoka, T., Takasaki, K. & Hanada, S. Carotenoids of Gemmatimonas aurantiaca (Gemmatimonadetes): identification of a novel carotenoid, deoxyoscillol 2-rhamnoside, and proposed biosynthetic pathway of oscillol 2,2'-dirhamnoside. *Microbiology* **156,** 757–763 (2010).

67. Al Atya, A. K. *et al.* Probiotic potential of Enterococcus faecalis strains isolated from meconium. *Front. Microbiol.* **6,** 227 (2015).

68. Dos Santos, K. M. O. *et al.* Safety, beneficial and technological properties of Enterococcus faecium isolated from Brazilian cheeses. *Braz. J. Microbiol.* **46,** 237–49 (2015).

69. Kundim, B. A. *et al.* New haliangicin isomers, potent antifungal metabolites produced by a marine myxobacterium. *J. Antibiot. (Tokyo).* **56,** 630–8 (2003).

70. Bouhired, S. M. *et al.* Biosynthesis of Phenylnannolone A, a Multidrug Resistance Reversal Agent from the Halotolerant Myxobacterium *Nannocystis pusilla* B150. *ChemBioChem* **15,** 757–765 (2014).

71. Gerth, K., Bedorf, N., Höfle, G., Irschik, H. & Reichenbach, H. Epothilons A and B: antifungal and cytotoxic compounds from Sorangium cellulosum (Myxobacteria). Production, physico-chemical and biological properties. *J. Antibiot. (Tokyo).* **49,** 560–3 (1996).

72. Daims, H. The Family Nitrospiraceae. in *The Prokaryotes* 733–749 (Springer Berlin Heidelberg, 2014). doi:10.1007/978-3-642-38954-2_126

73. Christensen, H., Kuhnert, P., Nørskov-Lauritsen, N., Planet, P. J. & Bisgaard, M. The Family Pasteurellaceae. in *The Prokaryotes* 535–564 (Springer Berlin Heidelberg, 2014). doi:10.1007/978-3-642-38922-1_224

74. Erlacher, A. *et al.* Rhizobiales as functional and endosymbiontic members in the lichen symbiosis of Lobaria pulmonaria L. *Front. Microbiol.* **6,** 53 (2015).

75. Dang, H., Li, T., Chen, M. & Huang, G. Cross-Ocean Distribution of Rhodobacterales Bacteria as Primary Surface Colonizers in Temperate Coastal Marine Waters. *Appl. Environ. Microbiol.* **74,** 52–60 (2008).

76. Oren, A. The Family Rhodocyclaceae. in *The Prokaryotes* 975–998 (Springer Berlin Heidelberg, 2014). doi:10.1007/978-3-642-30197-1_292

77. Brandl, H., Knee, E. J., Fuller, R. C., Gross, R. A. & Lenz, R. W. Ability of the phototrophic bacterium Rhodospirillum rubrum to produce various poly (beta-hydroxyalkanoates): potential sources for biodegradable polyesters. *Int. J. Biol. Macromol.* **11,** 49–55 (1989).

78. McIlroy, S. J. & Nielsen, P. H. The Family Saprospiraceae. in *The Prokaryotes* 863–889 (Springer Berlin Heidelberg, 2014). doi:10.1007/978-3-642-38954-2_138

79. Almeida, B. *et al.* Quantitative proteomic analysis of ibuprofen-degrading Patulibacter sp. strain I11. *Biodegradation* **24,** 615–630 (2013).

80. Cardman, Z. *et al.* Verrucomicrobia Are Candidates for Polysaccharide-Degrading Bacterioplankton in an Arctic Fjord of Svalbard. *Appl. Environ. Microbiol.* **80,** 3749–3756 (2014).

81. Sharma, V. *et al.* Metabolic and taxonomic insights into the Gram-negative natural rubber degrading bacterium Steroidobacter cummioxidans sp. nov., strain 35Y. *PLoS*

One **13,** e0197448 (2018).
